# High-Dose Convalescent Plasma for Treatment of Severe COVID-19

**DOI:** 10.3201/eid2803.212299

**Published:** 2022-03

**Authors:** Gil C. De Santis, Luciana Correa Oliveira, Pedro M.M. Garibaldi, Carlos E.L. Almado, Julio Croda, Ghislaine G.A. Arcanjo, Érika A.F. Oliveira, Adriana C. Tonacio, Dante M. Langhi, José O. Bordin, Renato N. Gilio, Leonardo C. Palma, Elaine V. Santos, Simone K. Haddad, Benedito P.A. Prado, Marjorie Cornejo Pontelli, Rogério Gomes, Carlos H. Miranda, Maria Auxiliadora Martins, Dimas T. Covas, Eurico Arruda, Benedito A.L. Fonseca, Rodrigo T. Calado

**Affiliations:** University of São Paulo, São Paulo, Brazil (G.C. De Santis, L.C. Oliveira, E.V. Santos, S.K. Haddad, B.P.A. Prado Jr., D.T. Covas, R.T. Calado);; Hospital Estadual de Serrana, Serrana, Brazil (P.M.M. Garibaldi, C.E.L. Almado);; Hospital Regional do Mato Grosso do Sul, Campo Grande, Brazil (J. Croda);; Fundação Oswaldo Cruz, Campo Grande (J. Croda);; Universidade do Mato Grosso do Sul, Campo Grande (J. Croda, G.G.A. Arcanjo);; Hospital São Camilo, São Paulo (É.A.F. Oliveira, A.C. Tonacio, D.M. Langhi Jr.);; Universidade Federal de São Paulo, São Paulo (D.M. Langhi Jr., J.O. Bordin);; Hospital Estadual de Américo Brasiliense, Américo Brasiliense, Brazil (R.N. Gilio);; Hospital das Clínicas da Faculdade de Medicina de Ribeirão Preto, Ribeirão Preto, Brazil (L.C. Palma);; Faculdade de Medicina de Ribeirão Preto da Universidade de São Paulo, São Paulo (M.C. Pontelli, R. Gomes, C.H. Miranda, M.A. Martins, E. Arruda, B.A.L. Fonseca)

**Keywords:** COVID-19, 2019 novel coronavirus disease, coronavirus disease, severe acute respiratory syndrome coronavirus 2, SARS-CoV-2, viruses, respiratory infections, zoonoses, convalescent plasma, neutralizing antibody, passive immunization, Brazil

## Abstract

To assess whether high-dose coronavirus disease (COVID-19) convalescent plasma (CCP) transfusion may benefit patients with severe COVID-19, we conducted a multicenter randomized trial in Brazil. Patients with severe COVID-19 who were within 10 days of initial symptom onset were eligible. Patients in the CCP group received 3 daily doses of CCP (600 mL/d) in addition to standard treatment; control patients received standard treatment only. Primary outcomes were death rates at days 30 and 60 of study randomization. Secondary outcomes were ventilator-free days and hospital-free days. We enrolled 107 patients: 36 CCP and 71 control. At day 30, death rates were 22% for CCP and 25% for the control group; at day 60, rates were 31% for CCP and 35% for control. Needs for invasive mechanical ventilation and durations of hospital stay were similar between groups. We conclude that high-dose CCP transfused within 10 days of symptom onset provided no benefit for patients with severe COVID-19.

Clinical signs and symptoms of coronavirus disease (COVID-19) are pleomorphic, varying from none (asymptomatic) to life-threatening. Typical signs/symptoms are fever, dry cough, dyspnea, fatigue, myalgia, anosmia, and ageusia (*1*). Radiography or computed tomography of the chest usually reveals bilateral pulmonary ground-glass opacifications, mainly in posterior and peripheral areas of the lungs (*2*). The most common laboratory test alterations are lymphopenia and elevated serum concentrations of inflammatory biomarkers and D-dimers (*3*). Risk factors for unfavorable outcomes are older age, concurrent conditions, and perhaps but of lesser importance, blood type A (*4*,*5*). Thus far, there is no consensual agreement about specific therapy for this disease, despite several attempts to develop one (*3*,*6*). More recently, antiviral agents such as MK-4482/EIDD-2801 and PF-07321332 seem to be promising (*7*,*8*).

In the past, passive antibody transfer by plasma or serum transfusion has been used clinically to treat other infectious diseases, including Ebola, influenza A, severe acute respiratory syndrome, and Middle East respiratory syndrome, as well as COVID-19 (*9*–*13*). The presence of antiviral antibodies, in patient serum or in COVID-19 convalescent plasma (CCP), has been associated with more favorable clinical outcomes (*14*). Thus, CCP seems to be an attractive therapy because it is a potential source of neutralizing antibodies (*15*,*16*).

The first case series reported from China suggested favorable outcomes for 5 patients receiving undergoing mechanical ventilation who received CCP on days 10–22 after hospital admission (*17*). Also in China, 10 critically ill patients received 200 mL of CCP with a neutralizing antibody titer of >640, which resulted in undetectable viral load and clinical improvement for 7 of the 10 patients (*18*). In a nonrandomized observational study that evaluated 3,082 CCP recipients, transfusion was associated with reduced mortality rates among patients who received CCP that had a higher titer against severe acute respiratory syndrome coronavirus 2 (SARS-CoV-2). Mortality rates within 30 days after CCP transfusion were 22.3% for the high-titer group, 27.4% for the medium-titer group, and 29.6% for the low-titer group. The relative risk for death was lower among patients who were not undergoing mechanical ventilation before transfusion (*19*). A prospective multicenter study in China that involved 103 patients with severe COVID-19 was stopped early, but initial findings suggested that CCP transfusion was associated with a higher percentage of patients being negative for SARS-CoV-2 by reverse transcription PCR (RT-PCR) at 72 hours (87.2%) than for controls (37.5%) (*20*).

Clinical improvement has been observed for Ebola patients with severe manifestations but not for those with life-threatening disease (*9*). Recently, a randomized trial in Argentina involving 228 patients who received CCP (median titer 3,200) and 105 who received placebo found that CCP transfusion did not reduce mortality rates at day 30 after randomization (10.96% for transfused and 11.43% for nontransfused groups) (*21*). A recent systematic review concluded that CCP transfusion makes little or no difference, at least for patients who needed mechanical ventilation (*22*).

To evaluate the efficacy and safety of high-dose CCP transfusion to treat severe COVID-19, we conducted an open-label multicenter randomized controlled trial. This study was approved by the national review board (Comissão Nacional de Ética em Pesquisa, CONEP; CAAE number 30509920.0.1001.0008). Written informed consent was obtained from all patients or legal representatives. The trial was performed in accordance with the principles of the Declaration of Helsinki and the International Conference on Harmonization–Good Clinical Practice guidelines. The trial was registered at the Brazilian Registry of Clinical Trials (http://www.ensaiosclinicos.gov.br, no. RBR-7f4mt9f).

## Materials and Methods

### Study Design

We conducted our investigator-initiated multicenter open-label randomized controlled trial in 5 hospitals: 4 in the state of São Paulo (Hospital das Clínicas da Faculdade de Medicina de Ribeirão Preto da Universidade de São Paulo, Hospital Estadual de Américo Brasiliense, Hospital São Camilo, and Hospital São Paulo); and 1 in Campo Grande, state of Mato Grosso do Sul (Hospital Regional de Mato Grosso do Sul). The 5 inclusion criteria were 1) diagnosis of COVID-19 based on RT-PCR results; 2) respiratory distress (oxygen saturation at room air <93%, or arterial partial pressure of oxygen/fraction of inspired oxygen <300, or requiring mechanical ventilation) resulting from pneumonia; 3) being within 10 days of initial symptoms; 4) age 18–80 years; and 5) signed written informed consent by the patient or legal representative. The 7 exclusion criteria were 1) history of previous severe allergy to plasma transfusion, 2) severe congestive heart failure, 3) terminal renal failure, 4) hepatic cirrhosis, 5) any severe illness expected to confer a short life expectancy, 6) participation in any other clinical trial with therapeutic intervention, and 7) immunosuppression. All included participants most likely had COVID-19 caused by the parental virus lineages (during the first wave), before emergence of the Gamma and Delta variants.

We enrolled 120 patients (40 in the CCP group and 80 in the control group), considering predicted death rates of 30% for the CCP group and 50%–60% for the control group. Computer-generated random numbering randomly assigned patients to receive either standard treatment (control) or CCP transfusion added to the standard treatment at a ratio of 2(control):1(CCP). For most patients, CCP transfusion was performed the day of or the day after randomization; only 2 patients received CCP 2 days after randomization. Patients and physicians were not blinded to treatment assignments. Placebo was not administered to control patients because we considered that the infused volume of saline or nonconvalescent plasma could harm the patients, especially those less tolerant to intravenous volume overload (i.e., those who were elderly, had acute kidney injury, or had other concurrent conditions). In addition, we considered it would be impossible to blind infusion of such a large volume of plasma to CCP patients. At the time of randomization, SARS-CoV-2 IgM/IgA was detected in all 65 patients who were tested and IgG was detected in 53 (81.5%).

### Convalescent Plasma Procurement and Transfusion

To prevent transfusion-associated lung injury, we limited CCP donor candidates to adult men or nulliparous women (*23*). According to regulation in Brazil, convalescent candidates may donate plasma after 15 days have passed since symptom resolution. Donor screening was similar to that used for conventional blood donation, including clinical evaluation for COVID-19 and access to peripheral veins. Plasma collection was performed by using a TRIMA ACCEL automated blood collection system (Terumo BCT, Inc., https://www.terumobct.com). We determined neutralizing antibody titers as described elsewhere (*24*). For both groups, transfused CCP median neutralizing antibody titer against SARS-CoV-2 was 128 (minimum titer of 64 in just 1 plasma unit). CCP units did not undergo pathogen reduction.

The total transfusion dose of CCP per patient was 1,800 mL (minimum dose 1,200 mL), divided into 3 daily doses of 600 mL for 3 days. The 600 mL volumes were divided into 2 subunits of 300 mL or 200 and 400 mL. All patients were randomized during days 7–10 after symptom onset, and the first CCP transfusion was administered on day 9 (range 8–10) for both groups. The first CCP transfusion had to be given by day 10 of initial symptoms.

We performed neutralizing assays for serum samples obtained from each plasma unit. In brief, we conducted virus neutralization testing with SARS‐CoV‐2 in 96‐well plates containing 5 × 10^4^ cells/mL of Vero cells (CCL‐81). Serum samples were initially inactivated for 30 min at 56°C. We used 11 serial dilutions (1:2 to 1:2,048). Subsequently, we mixed serum and virus (vol/vol) and preincubated the mixture at 37°C for 2 h for neutralization. We transferred the serum/virus mixture onto the confluent cell monolayer and incubated at 37°C at 5% CO_2_. After 3 days, we analyzed the plates by using light microscopy to determine presence/absence of cytopathic effect. Neutralizing antibody titer is described as the highest serum dilution that impeded cytopathic effect.

The primary clinical outcome was death rate at days 30 and 60 from the day of randomization. Secondary outcomes were ventilator-free days and hospital-free days on days 30 and 60 after randomization and adverse reactions to plasma transfusion. Adverse events were graded according to the Common Terminology Criteria for Adverse Events version 5.0.

### Patient Serologic Testing and Measurement of C-Reactive Protein and Interleukin-6

Using ELISA, we tested serum samples at randomization for the presence of SARS-CoV-2 IgM plus IgA (Vircell, https://www.vircell.com) and IgG (Euroimmun, https://www.euroimmun.com). We measured C-reactive protein (CRP) and interleukin 6 (IL-6) on days 0 and day 7 after randomization.

### Statistical Analyses

Results were expressed as mean ± SD or median (range) and proportions according to distribution characteristics. When comparing 2 groups, we used a 2-sided unpaired Student *t*-test (parametric data) or a Mann-Whitney test (nonparametric data). For statistical comparisons of categorical variables between groups, we used the χ^2^ test. We generated overall survival estimates by using the Kaplan-Meier method and assessed differences between the groups by using the log-rank test. We considered results to be statistically different when the p value was <0.05 (by 2-tailed testing). We used GraphPAD Prism version 8.4.3 (https://www.graphpad.com) for statistical analyses.

## Results

**Figure 1:**
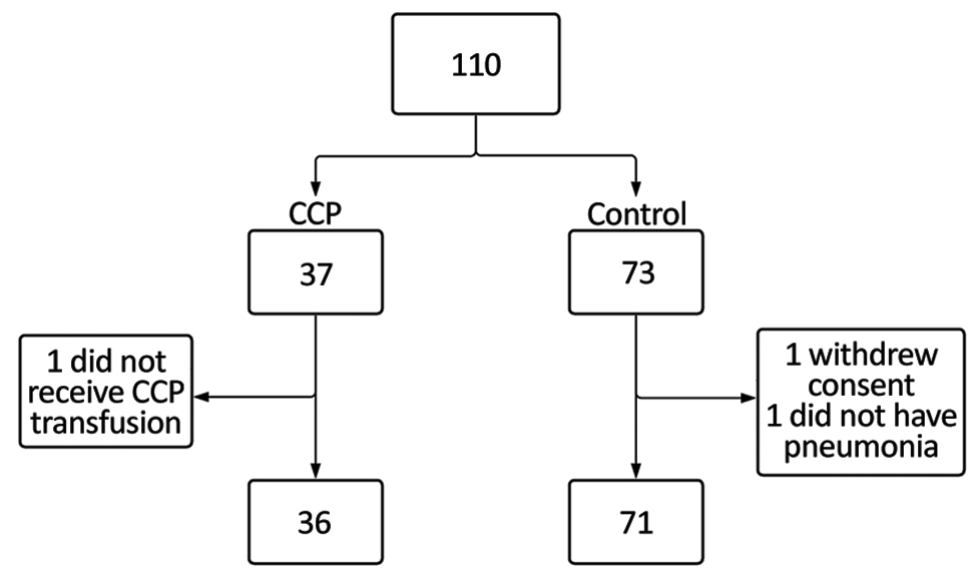
Enrollment and randomization process for study of high-dose CCP for treatment of severe COVID-19, Brazil. COVID-19, coronavirus disease; CCP, COVID-19 convalescent plasma.

During April–November 2020, we enrolled 110 patients at 5 centers. Because recruiting became more difficult as the number of new cases substantially decreased, we halted recruitment early, before reaching 120 participants. Of the 110, we excluded 3 participants from analysis: 1 in the CCP group did not receive plasma transfusion; 1 in the control group withdrew consent; and 1 in the control group was intubated and underwent invasive mechanical ventilation for neurologic reasons, not pneumonia, a prerequisite for inclusion in this study (Figure 1). The median duration of symptoms before randomization was 8 (range 7–10) days. The median age at randomization was 60 (range 24–80) years; male:female ratio was 1.7:1.0 (Table 1). All patients had severe COVID-19 (>6 points according to the World Health Organization severity ordinal scale (https://www.who.int/docs/default-source/documents/emergencies/minimalcoreoutcomemeasure.pdf).

**Table 1:** Baseline demographics and clinical characteristics of participants in study of high-dose convalescent plasma for treatment of severe COVID-19, Brazil*

Variable	CCP, n = 36	Control, n = 71	p value
Demographic			
Age, mean ± SD, y	56.11 ± 15.15	59.25 ± 12.35	0.25
Sex, no. (%)			
M,	23 (63.89)	44 (64.79)	1.0
F	13 (36.11)	27 (35.21)	
Body mass index, median (range), kg/m^2^	29.75 (18.37–58.00)	29.41 (20.31–74.22)	0.88
Weight, median (range), kg	85 (50–156)	85 (50–190)	0.95
Underlying conditions			
Hypertension, no. (%)	19 (52.78)	41 (57.75)	0.68
Diabetes mellitus, no. (%)	12 (33.33)	29 (40.85)	0.53
Renal replacement therapy, no. (%)	13 (36.11)	27 (38.03)	1.0
SAPS-3 score, median (range)†	56 (37–94)	68 (39–100)	0.15
SOFA score, median (range)	7.5 (1.0–14.0)	9.0 (2.0–14.0)	0.17
Clinical characteristic			
Mechanical ventilation, no. (%)	32 (88.88)	58 (81.69)	0.41
D-dimer, median (range), μg/mL‡	1.02 (0.27–10.00)	1.65 (0.39–20.00)	0.12
Blood type O/A§	13/18	31/27	0.38
Blood type, rH positive/negative§	33/3	67/3	0.41

*CCP, COVID-19 convalescent plasma; COVID-19, coronavirus disease; CRP: C-reactive protein; SAPS-3 score, Simplified Acute Physiology Score 3 at admission to intensive care unit; SOFA score, Sequential Organ Failure Assessment (on day of randomization) for 20 CCP and 41 control patients. †31 CCP and 57 control patients. ‡23 CCP and 39 control patients on day of randomization. §106 patients.

Because of low body weight (50 kg), 2 patients received a total of 1,200 mL of CCP. For 2 other patients, CCP doses were divided over 4 days, as allowed by protocol. No participant was unable to be reached during follow-up.

### Death Rates

**Figure 2:**
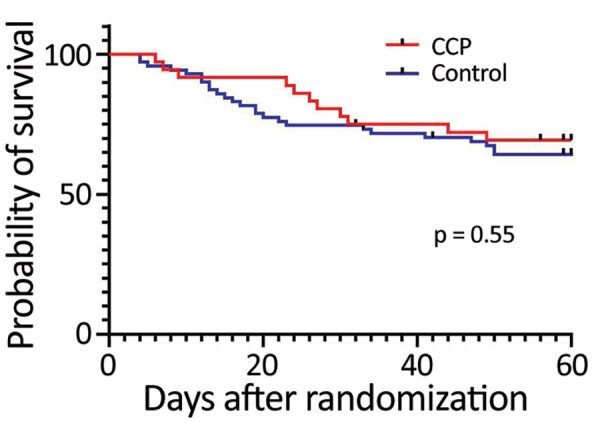
Probability of survival after randomization for study of high-dose CCP for treatment of severe COVID-19. COVID-19, coronavirus disease; CCP, COVID-19 convalescent plasma.

A total of 36 (34%) of the 107 enrolled patients died during hospitalization, 10 after day 30 (median 45.5, range 31–50 days); 3 were in the CCP group and 7 were in the control group (p = 1.00). At randomization day 30, death rates were 22% for the CCP group and 25% for the control group (odds ratio [OR] 0.84, 95% CI 0.32–2.25; p = 0.81). At day 60, death rates were 31% for the CCP group and 35% for the control group (OR 0.81, 95% CI, 0.35–1.86; p = 0.67) (Table 2). We performed a nonscheduled analysis of death rates on day 21 after randomization because at that point it seemed that there could be a difference between the groups, as suggested by the survival curve (Figure 2). We determined that on day 21, there had been a total of 3/36 (8.33%) deaths in the CCP group and 14/71 (19.7%) deaths in the control group (OR 0.37, 95% CI 0.11–1.3; p = 0.17).

**Table 2:** Clinical outcomes for participants in study of high-dose convalescent plasma for treatment of severe COVID-19, Brazil*

Outcome	CCP, n = 36	Control, n = 71	p value
Death at HD 30, no. (%)	8 (22.22)	18 (25.35)	0.81
Death at HD 60, no. (%)	11 (30.55)	25 (35.21)	0.67
Ventilator-free days at HD 30†	12.5 (0–30)	12.0 (0–30)	0.82
Ventilator-free days at HD 60‡	42.5 (0–60)	39.0 (0–60)	0.80
Hospital-free days at HD 30†	3 (0–24)	0 (0–28)	0.27
Hospital-free days at HD 60§	30.5 (0–53)	21.0 (0–58)	0.45

*CCP, COVID-19 convalescent plasma; COVID-19, coronavirus disease; HD, hospitalization day. †35 CCP and 70 control samples. ‡33 CCP and 67 control samples. §33 CCP and 69 control samples.

### Duration of Mechanical Ventilation and Hospitalization

At randomization day 30, the number of days free of invasive mechanical ventilation was 12.5 (range 0–30) for the CCP group and 12 (range 0–30) for the control group (p = 0.82); at day 60, the number of days was 42.5 (0–60) for the CCP group and 39 (0–60) for the control group (p = 0.80) (Table 2). We did not observe differences in hospital stay duration at days 30 and 60. At day 30, hospital-free days were 3 (0–24) days for the CCP group and 0 (0–28) days for the control group (p = 0.27); at day 60, hospital-free days were 30.5 (0–53) days for the CCP group and 21.0 (0–58) days for the control group (p = 0.43) (Table 2).

### Inflammatory Biomarkers

**Figure 3:**
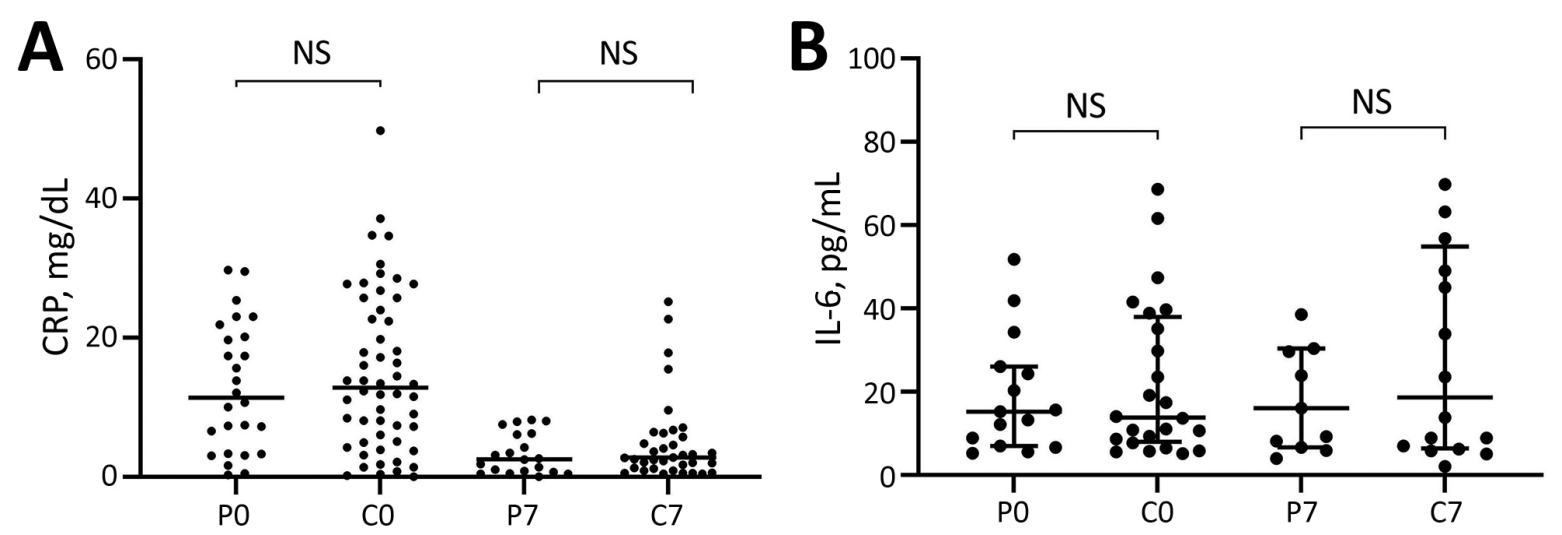
Scatter plots of inflammatory biomarker levels among participants in study of high-dose convalescent plasma for treatment of severe COVID-19, Brazil. A) C-reactive protein (CRP); total 80 patients (26 CCP, 54 control) on day 0 and 56 (20 CCP, 36 control) on day 7. B) Interleukin-6 (IL-6); total 39 patients (15 CCP, 24 control) on day 0 and 27 (11 CCP, 16 control) on day 7. Horizontal bars indicate medians. C0, control group day 0; C7, control group day 7; COVID-19, coronavirus disease; CCP, COVID-19 convalescent plasma; NS, not significant; P0, convalescent plasma group day 0; P7, convalescent plasma group day 7.

CRP concentrations were elevated at the time of randomization (day 0) and decreased significantly by day 7 in a similar fashion in both groups. The medians (interquartile ranges [IQRs]) on day 0 were 11.4 (3.31–20.55) mg/dL for the CCP group and 12.82 (5.05–24.40) mg/dL for the control group (p = 0.55). On day 7, medians (IQRs) were 2.53 (0.72–6.17) mg/dL for the CCP group and 2.75 (1.19–6.15) mg/dL for the control group (p = 0.52) (Figure 3, panel A). IL-6 concentrations were elevated on days 0 and 7 and, likewise, did not differ significantly between groups. IL-6 medians (IQRs) were 15.20 (6.99–26.00) pg/mL on day 0 and 13.80 (7.95–37.95) pg/mL on day 7 (p = 0.88) for the CCP group and 16.00 (6.61–30.40) pg/mL on day 0 and 18.65 (6.40–54.85) pg/mL on day 7 (p = 0.72) for the control group (Figure 3, panel B).

### Safety

No serious adverse reactions attributable to CCP transfusion were observed during study follow-up. We considered severe reactions to be greater than grade 3 according to the Common Terminology Criteria for Adverse Events version 5.0 (https://ctep.cancer.gov).

## Discussion

In this randomized clinical trial, transfusion of high-dose CCP did not reduce death rates, hospitalization durations, or number of days receiving mechanical ventilation for patients with very severe COVID-19. We detected a slightly reduced death rate, but it did not reach statistical significance. Serum inflammatory biomarkers were also reduced, but CCP transfusion did not influence the reduction. All enrolled patients experienced severe respiratory failure resulting from viral pneumonia, and most of them were undergoing invasive mechanical ventilation. Most patients had >1 concurrent condition, which increases mortality rates (*25*). More than one third of the enrolled patients needed kidney replacement therapy (hemodialysis). These characteristics emphasize the extreme severity of COVID-19 in the patients in our cohort. Participants received CCP as soon as possible, always within 10 days of symptom onset. This transfusion window was considered adequate at the time of the study planning and execution, especially when compared with other studies, in which transfusion occurred as late as day 39 (*9*). Of note, we observed that most trials evaluated the death rate at days 28 or 30 of randomization, but we observed that more than one fourth of the deaths in our study occurred during days 30–60.

Our results challenge those of nonrandomized studies previously conducted at the beginning of the pandemic (*17*), as well as those of a large nonrandomized study involving >3,000 US patients, which suggested that CCP could be an efficacious treatment for COVID-19 (*19*). In our study, mortality rate on day 30 was lower among patients who received CCP with higher titers of SARS-CoV-2 antibodies (22.3%) than among those who received CCP with medium (27.4%) or low (29.6%) titers. We observed a lower mortality rate for the high-titer group than for the low-titer group among patients who had not received mechanical ventilation before transfusion (relative risk 0.66, 95% CI 0.48–0.91) but not among patients who had received mechanical ventilation (relative risk 1.02, 95% CI 0.78–1.32) (*19*).

Our findings contrast with those of a previous multicenter randomized trial involving 103 participants (52 received CCP, 51 received standard treatment alone), which showed clinical improvement within 28 days in the subgroup of patients with severe disease who received CCP but not in the subgroup with life-threatening disease (*9*). In that study, CCP transfusion resulted in a higher rate of conversion to negative viral PCR results at 72 hours, suggesting potential benefit. In our study, most patients had life-threatening disease, which may explain, at least in part, the different outcomes. It is possible that patients with less severe disease may benefit from CCP. Nevertheless, in our study, an interim nonplanned analysis of death rates on day 21 suggested a possible benefit of CCP, similar to that observed by others (*26*–*28*), which was not confirmed by subsequent analyses. This finding raises the questions whether CCP provided a temporary benefit that was lost during the disease course and, if so, whether CCP should be transfused for a longer period during the disease.

Our study findings are in accordance with those of a randomized study in Argentina involving 228 patients who received CCP and 105 who received placebo, which did not show any survival benefits among patients receiving CCP (*21*). Of note, patient profiles for that study indicated less severe disease than did profiles for patients in our study. In the Argentina study, patients receiving mechanical ventilation were excluded, conflicting with the hypothesis that patients with less severe disease may benefit from CCP. The difference in disease severity also may explain the higher mortality rate observed in our study (33.64%) compared with that in the Argentina trial (10.96%). A study in Brazil also did not find clinical improvement in the group that received CCP (*29*). Our results are in agreement with those obtained in another randomized study, in which 464 participants with moderate COVID-19 were assigned to receive 2 doses of 200 mL CCP (n = 235) or standard treatment (n = 229) (*30*). The authors of that study evaluated the composite outcomes of progression to severe disease and observed that CCP transfusion was not associated with clinical benefit. A recent randomized clinical trial with >16,000 enrolled patients showed that CCP transfusion did not improve survival rates (*31*). In that trial, the 28-day mortality rate was 24% for both groups (1,399 of 5,795 vs. 1,408 of 5,763; p = 0.95). Also, CCP transfusion had no significant effect on the proportion of patients discharged from the hospital. In that trial, only 5% of the patients in each group were receiving invasive mechanical ventilation; however, this percent value meant that administration of CCP to >550 patients did not influence outcomes in that subgroup of patients. Last, a recently published multicenter randomized trial (patients hospitalized with moderate disease up to day 12 from symptom onset) also found that CCP did not reduce the risk for intubation or death at day 30 in hospitalized patients with moderate disease (*32*).

The first strength of our study is the randomized design, which provided homogeneity and adequate comparison between groups with similar characteristics and disease severity. Second, we used only CCP with adequate neutralizing antibody titers. Third, the transfused CCP volume was high, making it less likely that the lack of response could be attributable to a low dose of neutralizing antibodies. Fourth, the patients received CCP transfusion up to day 10 after symptom onset, which was relatively early in comparison with other studies (*9*,*17*). However, one may hypothesize that up to 10 days for CCP transfusion may be too late for those with the most severe disease. It is possible that by day 9–10 after symptom onset, most patients would have endogenous antibodies, which was determined for patients in our study and has been shown by others (A. Gharbharan et al., unpub. data, http://medrxiv.org/lookup/doi/10.1101/2020.07.01.20139857). Perhaps it would be more effective to administer CCP earlier in disease, especially for patients considered to be at higher risk for unfavorable outcome. Libster et al. recently demonstrated that early CCP transfusion (within 72 hours of symptom onset) in older patients with mild COVID-19 reduced progression to severe respiratory disease by 48% (*33*). Another group also demonstrated reduced hospitalizations for those who received early CCP transfusion with high titers of neutralizing antibodies (D.J. Sullivan, unpub. data, https://www.medrxiv.org/content/10.1101/2021.12.10.21267485v1). These results seem logical because a more effective clinical response with early CCP transfusion, before the spontaneous appearance of antibodies, would be expected.

Among the limitations of our study, the number of patients enrolled was relatively small. However, because we anticipated difficulties obtaining the necessary amount of CCP to be administered to each patient, we decided to assign the participants at a ratio of 2 control to 1 CCP. Another weakness was that the study was not blinded. However, infusion of a high volume of intravenous placebo could have been harmful to recipients. Patients in the control group should not be exposed to additional risk as a consequence of their participation in a clinical trial. Another limitation was that our patients already had SARS-CoV-2 antibodies when they received CCP transfusion, which could explain the absence of response to this therapy.

In conclusion, our study found that high-dose convalescent plasma transfusion provided no benefits for patients with severe COVID-19. Transfusions did not reduce death rates at days 30 and 60 from randomization, time receiving mechanical ventilation, or length of hospital stay for patients with severe COVID-19.
